# On the psychology of environmental preferences: The influence of contextual priming on discrete choice experiments

**DOI:** 10.1371/journal.pone.0312256

**Published:** 2024-10-31

**Authors:** Sandra Notaro, Petr Mariel, Constantinos Hadjichristidis

**Affiliations:** 1 Department of Economics and Management, University of Trento, Trento, Italy; 2 Department of Quantitative Methods, Faculty of Economics and Business, University of the Basque Country (UPV/EHU), Bilbao, Spain; Shimane Daigaku, JAPAN

## Abstract

This paper addresses an important gap in discrete choice experiments literature regarding the effect of contextual priming on preferences and willingness to pay. Contextual priming arises when the mere context in which a survey takes place–whether interviewees are approached in areas related or unrelated to the target issue under evaluation–can sway stated choices. We found priming to have a significant effect on one of the analyzed attributes associated with managing a natural park. We recommend interviewing participants in locations that are neutral with respect to the attributes under investigation. This procedure would prevent researchers from communicating incorrect recommendations to policymakers, natural resource planners, and managers.

## 1. Introduction

A challenge governments and international institutions face is how to allocate their limited resources. For example, what portion of its budget should a government allocate to improving the air quality in its big cities, improving education, or managing protected areas? This issue is complex because several goods of interest, such as environmental goods, are intangible and, therefore, do not have a market value. For such non-market goods, the regulatory bodies need a means to capture the value citizens place on them. These values, in turn, can inform political decisions that have palpable consequences for citizens. Two widely used methods to elicit such values are contingent valuation [1] (and discrete choice experiments (DCE) [2–4]. Both assume that people have stable preferences that can be elicited by asking the right questions in surveys [5].

In DCE, framing and priming refer to the observation that how information is presented to participants can influence their choices. Framing concerns how options or attributes of the choice problem are presented or framed. This can include the wording, the order in which options are presented, or the reference points used [6]. Priming refers to the influence of prior information on a participant’s choice, that is, how the situational context influences (passively and unintentionally) the accessibility of information that comes to mind and how this, in turn, influences how individuals think, feel, and behave [7]. Here, we examine the priming effect of whether the mere context in which a survey takes place–whether interviewees are approached in areas related or unrelated to the target issue under evaluation–can sway their preferences.

Psychological research has long demonstrated that preferences are not stable but constructed and that they can be influenced by aspects of the environment in which a response is elicited [8, 9]. The origins of priming research date back to the late sixties and were inspired by the spreading activation model of semantic memory, according to which the activation of a concept spreads it to other semantically related concepts [10]. For example, activating the concept of nature could also activate associated concepts with it in the memory, such as environmental protection.

There are different ways in which memory constructs can be primed, such as by having participants unscramble sentences connected to a particular theme like the environment [11] or by infusing the location where judgment takes place with a particular fragrance [12]. Here, we are interested in a natural and inescapable priming manipulation–the location where interviewees are surveyed. Consumer research has long recognized that the atmospherics of a retail shop, including the location, décor, sounds, aromas, and lightning, can systematically impact consumer behavior [13, 14]. Situational aspects are also central to nudging strategies [15]. For example, an effective way to gently prompt or “nudge” grocery shoppers into buying more healthy snacks is to position healthy snacks near the cash register [16]. However, location priming has received little attention in contingent valuation and in DCE studies on environmental goods, with a few exceptions [17, 18].

In the current study, we tested whether survey location influences stated preferences and willingness to pay (WTP) for the attributes of a natural park, which were elicited with a DCE. The survey was administered to park visitors, and the case study was of Monte Baldo Local Natural Park (MBLNP) located in northeastern Italy. Attributes were related to biodiversity protection (flora and fauna) and sustainable tourism development (trails and local food products).

The rest of the paper is organized as follows. In the second section, we review the literature on priming. In the third section, we present our methodology and then introduce and discuss the results in the fourth section. In the fifth section, we offer conclusions.

## 2. The priming effect

Priming concerns how context subtly and unobtrusively makes mental content accessible and the effect that this, in turn, can have on how people think, feel, judge, and behave [7]. Priming was inspired by theories of memory and, in particular, by the spreading activation model [10]. According to this model, knowledge can be schematically represented as a web diagram composed of nodes and links between nodes. The nodes represent concepts or cognitive units, and the links connect semantically associated concepts. Shorter links represent stronger associations between concepts. When a cognitive unit is activated, part of the activation passes through the links to other concepts, increasing their mental availability.

Early research on priming examined semantic priming with words. For example, participants were asked to identify as quickly and accurately as possible whether a string of letters presented on a screen did or did not form a word [19]. It was found that words such as “nurse” were more quickly recognized as words when they were preceded by related words such as “doctor” than when they were preceded by unrelated words such as “airplane,” thereby supporting semantic priming. Such paradigms have been used in cognitive psychology as a means of inferring the structure of semantic representations.

Since this early research, priming has captured the interest of social psychologists, who have demonstrated that it can affect the impressions we form of others [20] and even our behavior [21]. For example, in a pioneering set of studies participants were exposed to words connected to old age, such as Florida and bingo, and found that this made participants behave in a way consistent with the primed concept. Participants primed with old age walked slower to an elevator than those primed with an unrelated concept [21]. However, successive studies failed to replicate this finding [22].

Subsequent studies suggested that such failures were in part due to the presence of moderating factors that influence the magnitude and even the directionality of the priming effect. Specifically, they suggested that priming effects are rarely direct from prime to behavior, but rather they depend on a number of factors such as the attitudes people have towards the target stimulus [23, 24]. For example it was found that the impact of an elderly prime on behavior critically depends on the attitude people have toward the elderly [25]. For those with a positive attitude toward the elderly, pictures of the elderly had an impact in line with the primed concept (participants walked more slowly). However, for those with a negative attitude toward the elderly, the same prime influenced behavior in the opposite direction (participants walked more quickly). The authors suggested that people act similarly to people they like (*assimilation*) but dissimilarly to people they do not like (*contrast*).

From a similar perspective, several psychological mechanisms through which primes can affect behavior and the variables that can moderate specific paths were discussed [24]. The most direct mechanism is when a prime activates a construct in memory that directly activates behavior (e.g., priming old age makes people walk more slowly). A slightly less direct way is when a prime activates a construct in the memory that activates a goal that, in turn, drives behavior (e.g., priming old age activates the goal of either facilitating or hindering interaction depending on the attitudes people have toward the elderly, which then affects walking speed). Other indirect ways exist, such as when a prime acts by changing the way we perceive a target person, situation, or the self, which in turn affects behavior. For example, a prime that makes people construct a prisoner’s dilemma game as a competitive game ("Wall Street Game") or a cooperative game ("Community Game") can affect how they play it [26, 27]. Thus, although prime-to-behavior effects exist, it is challenging to make specific predictions because multiple moderators can influence their magnitude and directionality (for challenges and critiques of priming in social psychology research, readers can refer to the 2014 special issue of the journal Social Cognition, *What is “Social Priming”*?).

Beyond cognitive and social psychology, priming has also been shown to affect environmental choices. For example, people were primed with the concept of the environment by having them unscramble sentences that either contained words associated with the environment (e.g., green, ecological, earth, nature) or words unrelated to the environment [11]. The task was to choose between television sets that differed in several dimensions, including one related to the environment. The authors found that participants who were primed with the concept of the environment placed more weight on the environmental dimension than did their control counterparts who were primed with unrelated words. Further studies have suggested that priming acts through the activation of relevant values, such as the value of environmental protection.

Priming has also been shown to influence choices in stated preference valuation studies [28, 29]. For example, it was examined whether honesty priming would help reduce the hypothetical bias in a DCE study [29]. Hypothetical bias is the difference between the WTP for goods elicited by hypothetical methods and the value obtained through non-hypothetical methods [30]. Typically, participants report higher WTP values in hypothetical settings. The authors found that priming participants with the concept of honesty by having them unscramble sentences containing words related to honesty instead of neutral words reduced the hypothetical bias. The target good of interest was almond products that differed in attributes related to their environmental friendliness and price.

More recently, in a stated-preference study, a visual priming manipulation (a short cartoon video of a person taking a picture with a smartphone vs. a neutral condition in which this video was not shown) was crossed with a framing manipulation (the background in the video was either a pristine natural landscape [positive frame] or a polluted urban landscape [negative frame]) [28]. The authors found that the natural landscape made pro-environmental attitudes more salient (as evidenced by higher ratings of the attribute “ethical and environmental characteristics”). Furthermore, participants in the nature-priming condition were willing to pay a higher premium to buy an environmentally friendly smartphone than participants in the urban landscape or neutral conditions.

The mentioned studies used priming manipulations that are not always easy to implement. For example, asking participants to unscramble words just before the target task is difficult. However, visual priming could be implemented by changing, for example, the background of a questionnaire. Research suggests that priming can also spontaneously and naturally occur from the location of a survey. For example, it was shown that citizens voting in schools are more likely to fund a school initiative than citizens voting in other polling locations [31]. The study used data from the 2000 general elections in the state of Arizona, which included a ballot initiative that proposed increasing educational spending by raising the state tax from 5.0% to 5.6%. This initiative received more support from citizens voting in schools, and this effect remained statistically significant even after controlling for factors that could account for this difference, such as voters’ political preferences or distance from schools.

The role of situational aspects on behavior has long been recognized in consumer research. For example, in a seminal article [14]) it was highlighted the importance of the atmospherics surrounding a target consumer product, such as the location of the retail shop, the décor, aromas, and lightning, on consumer behavior. A similar point was made in another study [13] that further noted that aspects of a situation, such as the physical surroundings of a target consumer product, can influence consumption to a comparable or greater degree than individual characteristics of consumers, such as their gender, race, age, and intellect. Although these early studies did not specifically mention priming as the underlying mechanism, they emphasized the role of situational characteristics, including location, on behavior. Situational aspects are also central in nudging strategies [15]. Nudges refer to any aspect of the choice architecture that systematically and predictably alters behavior while retaining people’s freedom of choice without using economic incentives. For example, an effective nudge to increase healthy food consumption is to place healthy snacks in a prominent place in a grocery shop either at eye level or near the cash registers [16].

More pertinent to the present purposes, location priming has been shown to influence people’s tendency to engage in pro-environmental behaviors. For example, it was found that the building in which a behavior is observed can affect that behavior [32, 33]. The experimenters recorded how people disposed of their waste during lunchtime in an eating area in a building designed with sustainability in mind and in an eating area in a less green building. Responses to a questionnaire suggested that people who frequented the two sites were similar in terms of demographics. Both sites had a clearly marked disposal area with three types of bins: compost, garbage, and recycling. The experimenters found that people in the green building were more likely to correctly dispose of their waste than people in the less sustainable building.

Similarly, the present research investigates whether the location in which a survey takes place affects interviewees’ responses in choice tasks regarding different management actions in a nature park. To achieve this, we surveyed people in specific park locations, each related to a management action under evaluation. We predicted that when the survey location was semantically related (as opposed to unrelated) to the target management action, interviewees would give more weight to the attribute associated with that action.

Although we anticipated a priming effect, we had no predictions about its directionality. As previously mentioned, the magnitude and directionality of priming effects can vary depending on various characteristics. For example, although being interviewed close to trails could highlight the importance of trails and the need to conserve them, different individuals could have different beliefs about how this goal is best achieved. Some might value human intervention positively, while others might value it negatively. Furthermore, although being interviewed close to where a particular species lives could activate concepts related to that species, the impact of this activation on WTP judgments depends on the affective attitudes the interviewees have toward the species. Thus, although we anticipated that priming would influence preferences for management actions, we were mute about the directionality of this effect.

## 3. Materials and methods

### 3.1 The study area

Data for this case study are derived from a questionnaire to evaluate visitors’ preferences for management measures at a natural park, specifically, the Monte Baldo Local Natural Park, situated in the province of Trento in northeastern Italy. Rising straight from the Garda Lake, MBLNP covers 46.5 km^2^, from a few hundred meters above sea level to an altitude of over 2,000 meters. The park contains nine protected areas: five Natura 2000 areas and four regional or local reserves. The park is abundant in plant biodiversity. In particular, there are 28.7 species of flora per km², whereas other protected areas in the province of Trento have 2.3 species per km². Ten species of flora are protected by the European Union, and 60 species of wild orchids are present in the park. Thanks to its extraordinary biodiversity, this area has been a popular destination for naturalists, apothecaries, and pharmacists since 1400 and is known as “Hortus Italiae” (Garden of Italy). Several animal species live in the park, including rare reptiles and amphibians, such as the protected yellow-bellied toad (*Bombina variegata*).

MBLNP is one of the oldest parks in the reserve network (RN) of the Province of Trento. The RN was set up in 2008 as part of a European Project funded under the LIFE programme. The RN is a network of Natura 2000, regional or local protected areas, and interconnection zones managed to protect natural resources as well as to support the sustainable development of these areas, which are primarily dependent on agriculture and tourism [34]. RN is locally managed along with stakeholder participation under the supervision and coordination of the Province of Trento [35].

The name MBLNP has been used since 2013; the original name was the Brentonico Reserve Network. The distinction of a local natural park was given to this area because it meets specific naturalistic and territorial criteria required by the provincial law. The participation of local stakeholders is fundamental in managing the MBLNP, which is aimed at improving the sustainable development of agriculture and tourism while preserving biodiversity. An essential goal of the park is the protection and promotion of traditional activities, in particular, agriculture, for the benefit of sustainable tourism.

### 3.2 Survey design and administration

Four trained interviewers, two men and two women, aged between 24 and 26, collected data in on-site, face-to-face interviews between June 17 and September 9, 2017. Interviewing lasted all day on all weekends and two weekdays, which varied from week to week. A written informed consent was obtained from all subjects involved in the study.

Face-to-face interviews are advantageous to encourage the attention and effort of respondents. However, there are limitations to consider, such as a potential interviewer effect. Interviewers can clarify questions and explain more complex issues, but inadvertently, they can also be a source of measurement error [4]. To limit the interviewer effect, interviewers were instructed to minimize social interactions with respondents; the respondents filled in the questionnaires by themselves, while interviewers only explained the different sections of the questionnaire and assisted in filling out choice cards. In addition, on each interview day, the interviewers moved together to the different pre-identified locations in the park, changing the order of the locations each day so that they were not in the same location at the same time of the day. This precludes some systematic interviewer bias that could potentially arise if the different interviewers were assigned to collect data at different locations. We also trained the interviewers to standardize their behavior and asked for feedback during the entire time of the survey. All interviewers were dressed similarly: they wore a white T-shirt and jeans.

A systematic probabilistic sampling design was used to intercept respondents because there was no formal visitor list. Interviewers asked every second tourist they met to take part in the survey. People were always interviewed individually, even if they were part of a group. Both visitors and locals took part in the interviews, and the response rate was 65%.

The questionnaire was prepared following the guidelines for DCE [36, 37] and consisted of three sections. The first section contained warm-up questions and information about the RN and the actual management of the park. The second section included the illustration of attributes and levels. The survey’s core part was represented by choice cards, preceded by a script to ensure policy consequentiality [38]. This consequentiality script informed respondents that the results would be presented to the managers of Monte Baldo Local Natural Park and the Province of Trento and that they could be used to improve the management policies of the Park. Respondents were asked to answer as precisely as possible because the results must be accurate to aid policymakers in making the best decisions. We then asked respondents to pay attention to the cost, imagine that their choices were real, and imagine they would have to pay the price of the ticket on the day of the interview. We concluded by stating to participants that there were no correct or incorrect choices; we were just interested in their selections. The third part of the survey contained standard sociodemographic questions.

Relevant attributes were tested in a consultation process with experts and scientists, managers of the RN, managers of MBLNP, and naturalists. The initial set of candidate attributes resulted from lengthy discussions among local stakeholders regarding park management. From this list, four attributes were selected based on their importance in the local park management and the actual possibility of their implementation. Specialists and local stakeholders also determined the management measures associated with the specific attribute levels. The final set of attributes and levels are presented in Table 1.

**Table 1:** Attributes and levels.

Attributes	Description	Levels	No local management
**Biodiversity**	Conservation of biodiversity through the mowing of meadows and control of sheep grazing	1. Low (no action for biodiversity protection) 2. Medium (controlled sheep grazing) 3. High (mowing of meadows)	Low biodiversity of the meadows, no action to protect it
**Toad**	Protection of the yellow-bellied toad includes restoration and conservation of mountain puddles where the toad lives	1. Yes (action for toad’s protection) 2. No (no protection)	No protection
**Trails**	Restoration and improvement of the trails	1. No (no action) 2. Restoration (making trails safe and clean) 3. Restoration and enhancement (make the trails safe and clean and add signage; availability of paper and digital topographic maps)	No restoration or enhancement
**Organic products**	Availability of local organic products in farms, alpine huts, markets, and catering facilities	1. Yes (there are local products) 2. No (no local products)	No presence of local organic products
**Cost**	Price of daily entrance ticket to the park	€3, €6, €9, €12, €15, €18	€0

As shown in Table 1, the attributes for protection of the yellow-bellied toad and local organic food products have two levels, while biodiversity of the meadows and restoration and improvement actions on trails have three levels. The offered alternatives included different combinations of these levels along with the non-action alternative that represented the abandonment of local management in favor of centralized management by the Province of Trento. Central government management would imply no participation by local stakeholders and the impossibility of implementing actions specifically designed for local conditions. In fact, the Province of Trento would evenly undertake central management of natural areas, making it impossible to tailor management actions according to local environmental and socioeconomic conditions.

The monetary attribute was represented by an entrance ticket to the MBLNP, which would be necessary for the local community to co-fund local management initiatives. This attribute included six price levels based on the results of previous similar surveys conducted in the surrounding areas [39, 40]. Respondents faced 12 choice cards with three alternatives: two alternatives with a non-zero cost corresponding to options for local management and one non-action alternative representing the scenario in which the park is not locally managed. The non-action alternative was cost-free because this scenario did not include any investment in the described local management measures.

A pilot survey with 66 visitors was implemented on-site to test the design and wording of the survey. An Optimal Orthogonal Choice Design [41] was used to generate the choice cards in the pilot. The responses were used to set the prior values needed to generate an efficient design [42] for the final version of the survey. A sequential D-efficient design [43, 44] was used during the implementation of the survey by employing the parameter estimates of the first 383 questionnaires to further improve the efficiency of the design. The experimental designs were generated using NGene software [45].

Interviewers collected responses from 858 visitors. However, our final dataset consisted of 808 respondents, as 50 did not provide the complete sociodemographic information required for the analysis. We selected specific points inside the natural park to approach interviewees to test the priming effect. We interviewed people close to meadows to consider the priming effect of the meadows on flora biodiversity (149 respondents), close to mountain puddles where the yellow-bellied toad lives to test the contextual priming for the toad (59 respondents), close to mountain trails to test the priming effect for trails (322 respondents), and in or near huts and shelters where food was available to test priming for local organic products (218 respondents). Additionally, a control group of 60 respondents did not receive any treatment. These respondents were interviewed in a hotel in the center of the village of Brentonico. We interviewed people other than at mealtime to ensure the control group could not be subjected to priming. Unfortunately, the treatments are not balanced in terms of sample size because the location elements selected to induce priming are not present in the same quantity in the park and are not equally popular to tourists. Time and cost limitations during the data collection led to this relatively unbalanced number of respondents in different treatments.

### 3.3 Econometric analysis

Our econometric approach is pretty standard and is based on the random utility theory [46], with a linear function of the parameters’ utility defined as:

$$U_{int}=x_{int}^{\prime }\beta +\varepsilon_{int},$$ (1)

where *n* is the individual (*n* = 1,2,…,*N*); *i* is the alternative (*i* = 1,2,…,*J*); *t* is the choice situation (*t* = 1,2,…,*T*), *β* is a vector of the parameters; and *x*_*int*_ is a vector of the attributes. We opted for a latent class model (LCM) that addresses the issue of individual heterogeneity assuming a discrete mixing distribution for the parameters *β*, with individual parameters clustered in classes [47]. Given membership of class *c*, the probability of respondent *n*’s sequence of choices is given by:

$$P_{n|c}=\prod_{t=1}^{T}\frac{exp(x_{jnt}^{\prime }\;\beta_{c})}{\sum_{i=1}^{J}exp(x_{int}^{\prime }\;\beta_{c})}$$ (2)

The unconditional probability of choosing alternative *j* is a weighted average of all the parameter estimates *β*_*c*_ for each class *c*:

$$P_{n}=\sum_{c=1}^{C}\pi_{c}P_{n|c}=\sum_{c=1}^{C}\pi_{c}\prod_{t=1}^{T}\frac{\exp\left(x_{jnt}^{\prime }\;\beta_{c}\right)}{\sum_{i=1}^{J}\exp\left(x_{int}^{\prime }\;\beta_{c}\right)},$$ (3)

where *π*_*c*_ is the probability of belonging to the class *c*. The class allocation probabilities *π*_*c*_ are usually modeled using a logit structure, where the utility of a class is a function of the socio-demographic variables *SD*_*n*_ of the respondent and parameters *λ*_*c*_, in addition to a constant *μ*_*c*_:

$$\pi_{c}=\frac{\exp\left(\mu_{c}+{SD}_{n}^{\prime }\lambda_{c}\right)}{\sum_{i=1}^{J}\exp\left({\mu_{c}+SD}_{n}^{\prime }\lambda_{c}\right)}.$$ (4)

This model is estimated by the maximum likelihood method. The log-likelihood function to be maximized is defined as:

$$\log\;L=\prod_{n=1}^{N}\left[\sum_{c=1}^{C}\pi_{c}\prod_{t=1}^{T}\frac{exp(x_{jnt}^{\prime }\;\beta_{c})}{\sum_{i=1}^{J}exp(x_{int}^{\prime }\;\beta_{c})}\right].$$ (5)

Each attribute was interacted with a dummy variable indicating the corresponding priming stimulus to test if the priming effect produces differences in the respondent’s choices. Biodiversity of the meadows was thus interacted with being interviewed close to flowery meadows, protection of the toad with being interviewed close to mountain puddles, trails with being interviewed close to trails, and local organic products with being interviewed in or close to huts and shelters where food is available.

The LCM has been estimated using the Apollo package in R [48].

## 4. Results

Our aim is to examine whether the differences in preferences found between sites are the result of priming. It is possible that people with different recreational tastes and preferences choose different locations, creating an endogeneity problem. To address this, we provide a detailed breakdown by location of the descriptive statistics of the sociodemographic variables (age, gender, and education) used in the analysis. If there are no significant differences in these statistics, it is likely that the potential endogeneity problem can be considered negligible.

Tables 2 and 3 show the descriptive statistics for the sociodemographic variables analyzed. The average age of the respondents was 43.5 years, and almost half were male (49%). Regarding educational attainment, 52% of respondents had completed secondary education, and 38% had a university degree. These descriptive statistics are in line with the typical profile of tourists who visit the area under study [49].

**Table 2:** Descriptive statistics for the variable *Age*.

						*t-test*
	Mean	Median	Std. Dev	Min	Max	*p-value*
*Total*	43.5	44	13.9	17	80	
**Priming:**						
*Biodiversity*	45.2	46	13.9	28	74	0.06
*Toad*	43.9	43	11.9	19	74	0.03
*Trails*	42.1	41	14.8	17	77	<0.01
*Organic products*	42.9	43	12.5	18	80	0.01
*Control group*	49.1	49	13.1	23	80	

**Table 3:** Descriptive statistics for the variables *Gender* and *Education*.

			Number of observations				
			149	59	322	218	60
		*Total*	*Biodiversity*	*Toad*	*Trails*	*Organic*	*Control*
						*products*	*group*
**Gender**	*(Male = 0*,	49%	46%	39%	54%	47%	50%
	*Female = 1)*	51%	54%	61%	46%	53%	50%
	*p-value (χ² test)*		*0*.*74*	*0*.*31*	*0*.*66*	*0*.*82*	
**Education**	*Mandatory schooling*	10%	11%	3%	8%	10%	17%
	*Technical school*	10%	14%	5%	10%	8%	13%
	*High school diploma*	42%	42%	36%	43%	47%	27%
	*University degree*	31%	29%	46%	33%	28%	33%
	*Master’s or PhD degree*	7%	4%	10%	7%	7%	10%
	*p-value (χ² test)*		*0*.*13*	*0*.*05*	*0*.*10*	*0*.*06*	

By examining the *p-values* from the *t-test* for mean differences provided in Tables 2 and 3, it can be deduced that the means associated with various priming conditions (Biodiversity, Toad, Trails, and Organic products) significantly differ from those of the control group. By applying weights derived from entropy balancing [50], we reweighted our sample to match the control group on these covariates.

Entropy balancing, is a multivariate reweighting method designed to create balanced samples in observational studies [50]. This method ensures that the reweighted sample matches the characteristics of the control group across specified covariates, thereby mitigating endogeneity concerns. Entropy balancing operates by adjusting the weights of the treatment group observations so that the weighted sample moments (e.g., means, variances) of the covariates align with those of the control group.

Table 2 shows the analysis for the variable Age, and Table 3 for the variables *Gender* and *Education*. The variable Education had five levels: mandatory schooling (1), technical school (2), high school diploma (3), university degree (4), master’s degree, or PhD (5). As seen from Table 3, the *p-values* of the *χ² test* show that the frequencies of male and female participants are not different across the groups, with male participants making up around half of the respondents in each group. According to the *p-values* of the *χ² test* for differences in proportions, the participants’ educational level differs across the groups. The lowest *p-value* for the Toad group seems to be due to the relatively limited sample size of this group rather than to an endogeneity problem.

The control group was interviewed in a hotel, and to ensure that the location itself does not introduce other contextual influences, we included a more comprehensive analysis of the control group’s responses compared to the primed groups. Specifically, similar to Table 3, we analyze in Table 4 the proportions of responses. According to the *p-values* of the χ² test for differences in proportions, the participants’ responses differ between the *Control group* and the *Toad* and *Organic products* groups. Nevertheless, the proportions of the *Control group* and the *Biodiversity* and *Trails groups* do not differ. We would expect significant differences between the *Control group* and all other groups if some uncontrolled contextual influences were present in this group.

**Table 4:** Descriptive statistics for the variables *Choice*.

			Number of observations				
			149	59	322	218	60
		***Total***	*Biodiversity*	*Toad*	*Trails*	*Organic*	*Control*
						*products*	*group*
**Choice**	*Alternative 1*	66%	66%	80%	67%	63%	60%
	*Alternative 2*	24%	24%	12%	26%	21%	35%
	*Alternative SQ*	10%	10%	8%	6%	16%	5%
	*p-value (χ² test)*		*0*.*19*	*0*.*01*	*0*.*38*	*0*.*02*	

Given that other contextual factors might also influence the results, we have included a dummy variable, *Sunny and not windy day*, representing the weather conditions in the model. Its value equal to one represents a sunny day (62.1%), while zero represents partly sunny, mostly cloudy, cloudy, and rainy days (37.9%).

The specification of the number of classes in an LCM is not integrated into the maximum likelihood criterion. Instead, it is typically established through the utilization of information criteria. Table 5 presents the Akaike Information Criteria (*cAIC* and *AIC*), Bayesian Information Criteria (*BIC*), and log-likelihood values (*LogL*) for two- and three-class LCM.

**Table 5:** Information criteria.

	2 Classes	3 Classes
*LogL*	-14,619.3	-13,836.9
*Number of parameters*	35	55
*Sample size*	9,696	9,696
*AIC*	29,308.5	27,783.9
*AIC3*	29,343.5	27,838.9
*BIC*	29,559.8	28,178.8
*CAIC*	29,594.8	28,233.8

An increased number of classes gave rise to numerical optimization challenges during the estimation process, primarily due to flat regions within the log-likelihood function. These flat regions rendered unfeasible the estimation of LCM with a higher number of classes. According to some authors [51, 52], the statistical criteria and the significance of the parameter estimates need to be tempered by the researcher’s own judgment of the suitability of the model when the number of classes is determined, and we, therefore, estimated a three-class model.

Table 6 presents the estimates of the three-class LCM.

**Table 6:** Results of the latent class model.

	Class 1			Class 2			Class 3		
	Coeff.	Rob t		Coeff.	Rob t		Coeff.	Rob t	
Attributes									
*ASC1*	2.75	5.78	***	1.95	7.28	***	0.70	0.69	
*ASC2*	2.59	5.32	***	1.95	7.18	***	0.66	0.55	
*Biodiversity Medium*	0.71	6.92	***	0.58	6.43	***	0.75	2.46	**
*Biodiversity High*	0.81	7.54	***	0.86	7.79	***	0.73	2.65	***
*Toad*	0.62	6.33	***	1.18	8.93	***	0.96	2.50	**
*Trails Medium*	1.47	8.61	***	1.30	10.84	***	1.39	3.02	***
*Trails High*	1.91	7.77	***	1.62	8.39	***	1.56	1.31	
*Organic products*	0.69	7.49	***	0.76	7.74	***	0.96	4.85	***
*Cost*	-0.24	-13.17	***	-0.05	-2.54	**	-0.45	-3.99	***
**Priming effect**									
*Biodiversity Medium*	-0.09	-0.19		-0.08	-0.58		-0.22	-0.41	
*Biodiversity High*	-0.19	-0.50		0.07	0.48		-0.20	-0.16	
*Toad*	-0.44	-1.18		-0.17	-0.77		1.02	1.15	
*Trails Medium*	0.10	0.41		-0.13	-0.92		-0.33	-0.75	
*Trails High*	-0.11	-0.36		-0.19	-1.00		-0.41	-0.70	
*Organic products*	0.16	1.04		0.34	2.61	***	-1.19	-3.49	***
**Class allocation parameters**									
*Constant*				-0.33	-0.52		-0.84	-0.79	
*Woman*				0.23	0.91		0.11	0.28	
*Age*				0.02	1.65	*	-0.01	-0.53	
*Education*				-0.16	-1.14		-0.09	-0.32	
*Sunny and no windy day*				0.27	0.30		0.27	0.68	
**Class probability**									
*Class 1*	0.37								
*Class 2*	0.52								
*Class 3*	0.11								
**Log-likelihood**	-13,836.94								
**Number of parameters**	55								
**Observations**	9,696								
**AIC**	27,783.88								
**BIC**	28,178.75								

***, **, *: significance at the 1%, 5% and 10% level

The first block (Attributes) includes the estimates of the attribute coefficients *β*_*c*_ defined in (2). The second block (Priming effect) presents the coefficients of the interactions of the attributes with the corresponding priming effects. The third and fourth blocks (Class allocation parameters and Class probabilities) of Table 6 present the estimates of the parameters of the allocation function defined in (4) and the mean allocation probabilities, respectively. Parameters *μ*_1_ and *λ*_1_ in (4), corresponding to the first class, were set to zero to ensure the identification of the model. The vector of sociodemographic variables *SD* defined in (4) includes gender, age, and education level.

The first conclusion that can be drawn from Table 6 is that the positive values of the alternative specific constants (*ASC*s) representing the two alternatives associated with local management related to an additional payment indicate a general interest in the local management measures for MBLNP. These constants are significant at 5% in the two largest Classes 1 and 2. In all three classes, all estimated coefficients of the main effects of the non-cost attributes have the expected positive signs, and they are statistically significant at 5%. As expected, the cost coefficient is negative in all classes, indicating a decreasing marginal utility to the price of the park entrance fee.

Based on the LCM estimates in Table 6, we derived the WTP for each attribute in the estimated three classes. These class-specific WTP values with and without priming treatment interaction are presented in Table 7.

**Table 7:** Class-specific WTP values without and with priming.

	Class 1	Class 2	Class 3
	*Without priming effect*		
*Biodiversity Medium*	2.9 €	13.0 €	1.7 €
*Biodiversity High*	3.4 €	19.1 €	1.6 €
*Toad*	2.6 €	26.3 €	2.2 €
*Trails Medium*	6.1 €	28.9 €	3.1 €
*Trails High*	7.9 €	35.8 €	3.5 €
*Organic products*	2.9 €	17.0 €	2.1 €
	*With priming effect*		
*Biodiversity Medium*	2.6 €	11.1 €	1.2 €
*Biodiversity High*	2.6 €	20.7 €	1.2 €
*Toad*	0.7 €	22.5 €	4.4 €
*Trails Medium*	6.5 €	26.1 €	2.4 €
*Trails High*	7.5 €	31.6 €	2.6 €
*Organic products*	3.5 €	24.6 €	-0.5 €

Considering the estimates in Table 6 and the WTP values in Table 7, we can characterize the classes accordingly. The largest Class 2 stands out due to its notably high WTP values, primarily for the restoration and improvement of trails. In contrast, the second-largest Class 1 exhibits approximately four times lower WTP values across all attributes, except for Toad, which is nearly ten times lower. The smallest Class 3 presents WTP values roughly ten times lower than those in Class 2. What sets these two classes apart is the presence of a significant priming effect related to Organic products. While this attribute is highly regarded in Class 2, it is not favored in Class 3. Consequently, the modeled preference heterogeneity in this LCM reveals a distinct and opposing priming effect, serving as the defining feature between Class 2 and Class 3. It is important to highlight that an LCM is not a classification method because class membership is probabilistic. The key objective is to compute the final WTP values, which are derived as weighted means of the WTP within each class; an LCM is a tool to approximate the underlying preference heterogeneity. LCM has the advantage of being a semiparametric specification that alleviates the need for potentially stringent or unwarranted distributional assumptions regarding individual heterogeneity, which are needed, for example, in a Random Parameter Logit model.

To analyze the priming effect on the WTP values in greater detail, their values for individual *n* are computed as the average of the ratio non-cost/cost coefficients, weighted by the probability defined by the allocation function (4). If *β*_*cr*_ is the coefficient of the *r*-th non-cost attribute (*r* = 1,2,…,6) in class *c* and *β*_*c*7_ is the cost attribute in class *c*, then the individual WTP values in our three-class case can be defined as:

$${WTP}_{nr}=\pi_{n1}\frac{\beta_{1r}}{\beta_{17}}+\pi_{n2}\frac{\beta_{2r}}{\beta_{27}}+\pi_{n3}\frac{\beta_{3r}}{\beta_{37}}.$$ (6)

**Fig 1:**
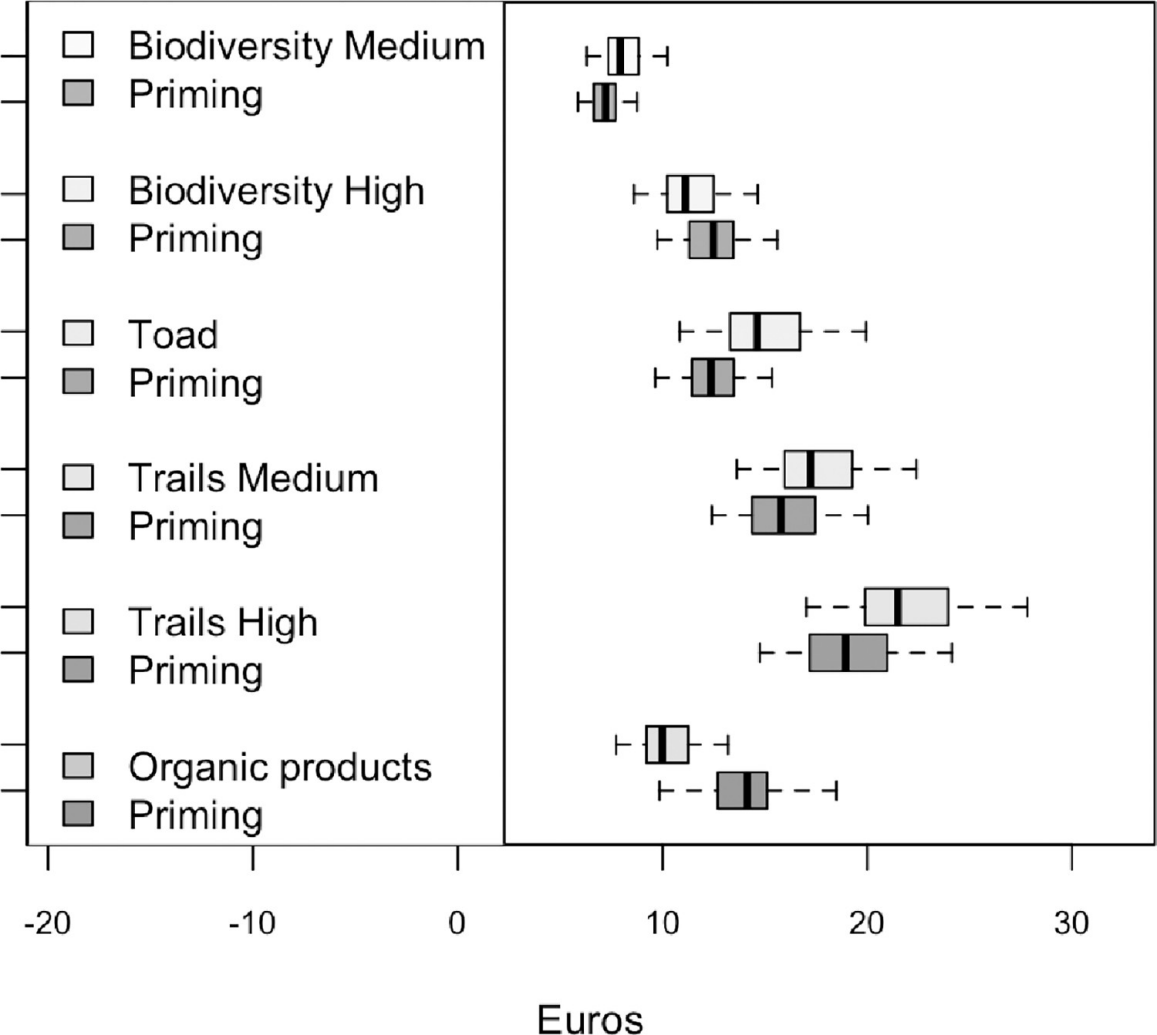
Distributions of individual WTP values.

Table 8 presents the descriptive statistics of the WTP distributions based on the estimates included in Table 6. Fig 1 graphically presents the same information.

**Table 8:** Descriptive statistics of the individual WTP values.

	Mean	Minimum	1st Quart.	3rd Quart.	Max.
	*Without priming effect*				
*Biodiversity Medium*	8.1 €	6.3 €	7.4 €	8.8 €	10.2 €
*Biodiversity High*	11.1 €	8.6 €	10.2 €	12.5 €	14.7 €
*Toad*	15.0 €	10.8 €	13.3 €	16.7 €	20.0 €
*Trails Medium*	17.6 €	13.6 €	16.0 €	19.3 €	22.4 €
*Trails High*	21.9 €	17.0 €	19.9 €	24.0 €	27.8 €
*Organic products*	10.2 €	7.7 €	9.2 €	11.3 €	13.2 €
	*With priming effect*				
*Biodiversity Medium*	7.2 €	5.9 €	6.7 €	7.7 €	8.8 €
*Biodiversity High*	12.5 €	9.8 €	11.3 €	13.5 €	15.6 €
*Toad*	12.6 €	9.7 €	11.4 €	13.5 €	15.4 €
*Trails Medium*	16.0 €	12.4 €	14.4 €	17.5 €	20.0 €
*Trails High*	19.1 €	14.8 €	17.2 €	21.0 €	24.1 €
*Organic products*	14.1 €	9.9 €	12.7 €	15.1 €	19.2 €

The differences between two WTP distributions corresponding to WTP with and without priming effect are tested by the complete Poe’s combinatorial test [53], which is a one-tailed test for the null hypothesis of equality of the tested distributions. The results of this test are shown in Table 9.

**Table 9:** Poe test for the difference of WTP distributions without and with priming.

Attribute level	H_a_	*p-value*
*Biodiversity Medium*	*WTP (no priming) > WTP (priming)*	0.24
*Biodiversity High*	*WTP (no priming) < WTP (priming)*	0.29
*Toad*	*WTP (no priming) > WTP (priming)*	0.18
*Trails Medium*	*WTP (no priming) > WTP (priming)*	0.29
*Trails High*	*WTP (no priming) > WTP (priming)*	0.22
*Organic products*	*WTP (no priming) < WTP (priming)*	0.05

The core of our study is related to the analysis of priming effects. Focusing on Fig 1 and Table 9, only *Organic products* shows a positive and statistically significant priming effect at a 5% level. Respondents interviewed in or near to huts and shelters where food was available showed higher WTP for organic products. The mean value of the distribution for these respondents was €14.0, whereas for the control group it was €10.5. Food availability thus proved to be a strong positive stimulus for the respondents.

## 5. Conclusions

Methods of eliciting stated preferences are founded on the assumption that individuals are rational and have stable preferences. However, the psychological literature has shown that preferences are unstable and can be influenced by aspects of the context in which a survey is implemented [8]. The situational context can influence the accessibility of information that comes to mind, including thoughts, feelings, goals, behaviors, and preferences. This aspect can be an element of context-dependence in non-market valuation, violating the assumption of the stability of preferences and biasing welfare estimates. In our study, we tested the effect of the location where people were interviewed, exploring whether locations related to some attribute of the choice experiment influenced choices for that attribute. Our results showed a positive priming effect for the attribute organic products, while no effect for the other attributes.

But why was there a priming effect for organic products, but not for other attributes? We believe that several factors must be present for priming effects to occur: (1) the prime should be potent enough to activate relevant mental constructs in memory (necessary condition), (2) people should have strong and uniform attitudes towards the target good, and (3) people should have strong and uniform attitudes towards the suggested interventions to improve the provision of the target good [23, 24]. Below, we detail these factors and argue that they were likely all present only for organic products.

A prime acts by activating concepts, values, and goals in the memory. Therefore, a precondition for priming to occur is the ability of the prime to activate the relevant mental constructs. For example, it is likely that interviewing people in restaurants would make salient the concept of food (and organic products) and interviewing people close to trails would make salient concepts related to hiking. However, interviewing people close to the habitat of the yellow-bellied toad (especially outside the toad season) would be a less potent cue for activating related mental concepts.

Second, even if a location provides a strong prime, the strength and direction of the priming effect can still vary. One determining factor is the attitude people have towards the target good (positive/negative) and how consistent it is across people [25]. For instance, people are more likely to have a uniformly positive attitude towards organic products, while their attitude towards the yellow-bellied toad could vary. Some individuals may have a positive feeling toward the yellow-bellied toad, while others may have negative feelings. Studies have shown that people’s emotions and values towards wildlife-related can vary significantly [54–56]. Therefore, even if a location provides a potent cue for activating relevant mental constructs, the strength and the directionality of the priming effect can vary. When people’s attitudes towards a target are uniform and strong, then the priming effects should be either strongly positive or strongly negative. When peoples’ attitudes are more heterogeneous, the net priming effect would be null or weak.

A third factor that may influence priming effects is the nature of the interventions mentioned in the levels of provision. For example, for Organic products, there were only two levels of provision: either these products were available or not. Therefore, to the extent that people have a positive attitude towards organic products, they are likely to favor their availability, resulting in a positive priming effect. However, for other target goods such as trails or biodiversity, the interventions were more complex. For example, trail intervention included levels such as restoration and enhancement, whereas biodiversity included different levels of human involvement (controlled sheep grazing versus mowing). Even if people have a uniformly positive attitude towards these goods, they might disagree about how to implement changes. Some people might favor human interventions (e.g., increasing biodiversity by mowing the grass rather than letting cows graze on it; improving trails by adding signage and having paper and digital topographic maps rather than simply making trails safe and clean), while others can be against them. These opposing views concerning implementation can weaken a priming effect even if the location prime is potent and people have a uniformly positive or negative attitude toward the good.

It is important to recognize that the results of our study could be influenced not only by the inherent characteristics of the survey locations but also by the self-selection of participants. It is possible that the locations themselves, such as meadows, puddles, mountain trails, food pavilions, and a hotel, influenced participants’ responses due to their unique environmental contexts and appeal. Additionally, the self-selection bias, where participants choose their destinations based on personal preferences, may lead to an overrepresentation of individuals who highly value certain locations, thus impacting the survey outcomes. This dual influence of location characteristics and participant self-selection necessitates caution in interpreting the results, as the observed effects may reflect both the environmental context and the heterogeneous value individuals place on these areas within the park. Additional contextual factors, such as the specific time of day or day of the week when interviews took place, may have affected the results.

An additional limitation of this study is that the hotel setting can introduce several potential contextual influences that could bias the results. Different types of hotels might attract particular socio-demographic groups. For instance, luxury hotels may attract wealthier individuals, while budget hotels may appeal to those with lower economic status. Socioeconomic status, in turn, can directly influence WTP. The type of hotel (luxury vs. budget) could also have an indirect influence on guests by setting certain expectations and moods, which might influence how they perceive and value environmental attributes [57, 58]. Moreover, hotels located near major attractions might attract guests more interested in those specific attractions, which can be related to some of the analyzed environmental attributes. Additionally, proximity to natural or environmentally significant sites might attract guests who are more environmentally conscious, thereby influencing their responses. However, hotels might also attract individuals for different reasons than the target attributes (such as extreme sports), which may lead to decreased WTP for the target attributes.

To mitigate these effects, future studies should conduct surveys across a variety of hotel types and price ranges to capture a diverse range of economic backgrounds and minimize bias from any one type of hotel. Furthermore, surveys should be conducted at different times of the day and on different days of the week to reach a broader range of respondents and avoid temporal biases. Finally, surveys could ex post include debriefing questions to ask respondents if they believed that the survey setting influenced their responses.

Finally, like many survey-based studies, the current research relies on self-reported data, which can be subject to several biases such as the social desirability bias (respondents may provide answers that they believe are socially acceptable or favourable rather than their true feelings or behaviors), the recall bias (respondents may not accurately remember past behaviors or experiences) or the acquiescence bias (a tendency for respondents to agree with statements or questions regardless of their actual opinions). Although face-to-face interviews help mitigate some of these issues, they cannot eliminate them entirely. It is recommended to apply some of the possible strategies to tackle this problem, such as anonymity assurance, careful design of questionnaires, recall aids (timelines, calendars), conducting pilot studies, or collecting data through multiple methods or sources [59].

We know of very few studies that have used a DCE to investigate the effect of location on stated preferences [17, 18]. Tinch and colleagues investigated the effects of variations in the timing and location of choice experiment questions about conserving a UK national park. The same participants responded to the same choice scenarios on four different occasions: off-site just before visiting the park, on-site, off-site immediately after the visit, and off-site four months later. They found that participants gave very different answers during the on-site visit than in any off-site conditions (these took place in a community center). In particular, the on-site visit increased the variance of the error term (participants found it harder to choose between the alternatives) and removed attention to the price associated with each alternative. In our study, all the treatments of interest were on-site but in different locations. We found that for one particular target good (organic products) the on-site location in which participants were surveyed mattered: participants were more WTP when surveyed in the location that was most associated with the target product (e.g., a restaurant) than in other on-site locations.

The role of interview location on travel-time estimates has been investigated by interviewing participants using an internet panel, an email register, or during an actual journey [17]. It was found that those who answered while traveling assigned, on average, higher values to travel time than those who did not. The researchers suggested that this could be because the benefits of saving time are more salient when traveling. Our study and the proposed explanation are similar in that we also believe that location can affect choices by making certain associated characteristics more salient. However, we manipulated the physical location rather than the type of activity (travel) during the survey. In addition, when traveling, people may consciously consider the benefits of saving time, whereas in our case, the impact of location may have been more subtle. We also drew links between such effects and location priming, which was not considered in these previous papers.

Governmental agencies and international institutions spend millions to survey people’s preferences. These surveys are thought to reveal people’s real preferences about the target issues and thus provide input for litigation and political decisions and, ultimately, to guide policy. One of the best-known examples was the assessment of natural resource damage due to the Exxon Valdez oil spill in Prince William Sound (Alaska) in 1989. The estimation was used in formulating the claim for compensation to the court in the case of the State of Alaska vs. Exxon [60]. We provide preliminary evidence that people’s responses in surveys may be systematically swayed by the subtlest of cues: the location where the interviewees happened to be surveyed.

Since our results confirm the influence of priming on stated preferences, practitioners must be cautious when designing surveys for environmental valuation. For instance, if a survey about conservation funding is conducted in a setting with a potent cue for activating related mental constructs, respondents might be more inclined to support higher funding due to the positive priming effect. Concretely, a survey estimating the WTP for the protection of endangered species might produce more accurate responses, if conducted in a neutral location as opposed to a nature reserve, where the presence of animals could influence responses. Similarly, a survey estimating the WTP to improve water quality may produce more accurate WTP if respondents are not surveyed nearby water bodies. To obtain more accurate valuation estimates, surveys should ideally be conducted in neutral locations to minimize the influence of environmental cues. This is important to ensure that public funding is allocated based on representative WTP estimates.

Nevertheless, finding truly neutral locations can be challenging and may require pre-testing and focus group discussions to identify areas that do not prime respondents towards certain attitudes. This can increase the logistic complexity of the survey implementation and its economic cost. It is noteworthy to mention that locations are not either neutral or not neutral in an absolute sense, but rather their neutrality depends on the attributes being studied. For example, although a mid-range hotel may provide a fairly neutral context for evaluating preferences towards an animal in danger, it might be non-neutral with regards to evaluating interventions that support touristic activities. This adds to the complexity of identifying neutral contexts.

In summary, recognizing and mitigating priming effects in environmental valuation and broader social research can lead to more accurate data collection and better-informed policy decisions. By identifying and implementing neutral survey locations and considering demographic variations, researchers can enhance the reliability and relevance of their findings and policymakers may allocate funds based on representative findings.

The present research has highlighted many questions in need of further investigation. Priming effects appear to depend on a number of factors, including the ability of a prime to activate relevant mental constructs, the attitudes people have toward the target good and the uniformity of such attitudes across people, as well as the attitudes people have toward the suggested interventions (levels of provision). To get a clearer picture of the mechanisms underpinning priming effects, future studies could include measures of these factors (strength of a prime, attitudes towards target good, attitudes towards proposed interventions) and measure their impact on responses. The findings of these studies could help refine current theoretical models and enable them to capture context effects.

## Supporting information

S1 Questionnaire (DOCX)

S1 File Design of the choice experiment. (PDF)
