# Supplementary material for: On the psychology of environmental preferences: The influence of contextual priming on discrete choice experiments
Source: PLoS One. 2024 Oct 31;19(10):e0312256. doi: 10.1371/journal.pone.0312256 (PMC11527292; doi:10.1371/journal.pone.0312256)
Supplement: S1 File — (PDF) [file pone.0312256.s002.pdf]

Design of the choice experiment

- The initial set of candidate attributes resulted from lengthy discussions among local stakeholders regarding park management.
- Specialists and local stakeholders also determined the management measures associated with the specific attribute levels.
- From this list of attributes, four attributes were selected based on their importance in the local park management and the actual possibility of their implementation.
- Relevant attributes were tested in a consultation process with experts and scientists, managers of the RN, managers of MBLNP, and naturalists.
- The questionnaire was prepared: The first section contained warm-up questions and information about the RN and the actual management of the park. The second section included the illustration of attributes and levels. The third part of the survey contained standard sociodemographic questions.
- A pilot survey with 66 visitors was implemented on-site to test the design and wording of the survey.
- An Optimal Orthogonal Choice Design was used to generate the choice cards in the pilot.
- The responses of the pilot were used to set the prior values needed to generate an efficient design for the final version of the survey.
- A sequential D-efficient design was used during the implementation of the survey by employing the parameter estimates of the first 383 questionnaires to further improve the efficiency of the design.
- Four trained interviewers, two men and two women, aged between 24 and 26, collected data in on-site, face-to-face interviews between June 17 and September 9, 2017. Interviewing lasted all day on all weekends and two weekdays, which varied from week to week.
- A systematic probabilistic sampling design was used to intercept respondents because there was no formal visitor list. Interviewers asked every second tourist they met to take part in the survey. People were always interviewed individually, even if they were part of a group.
- Interviewers collected responses from 858 visitors.
- We selected specific points inside the natural park to approach interviewees to test the priming effect:
    - Close to meadows to consider the priming effect of the meadows on flora biodiversity (149 respondents),
    - Close to mountain puddles where the yellow-bellied toad lives to test the contextual priming for the toad (59 respondents),
    - Close to mountain trails to test the priming effect for trails (322 respondents)
    - In or near huts and shelters where food was available to test priming for local organic products (218 respondents).

- A control group of 60 respondents did not receive any treatment. These respondents were interviewed in a hotel in the center of the village of Brentonico. We interviewed people other than at mealtime to ensure the control group could not be subjected to priming.
